# Evaluating the effectiveness of learning ear anatomy using holographic models

**DOI:** 10.1186/s40463-020-00458-x

**Published:** 2020-08-19

**Authors:** Joshua J. Gnanasegaram, Regina Leung, Jason A. Beyea

**Affiliations:** 1 Queen’s University School of Medicine, 15 Arch Street, Kingston, K7L 3N6 Canada; 2 Department of Otolaryngology, Kingston Health Sciences Centre, Queen’s University, 144 Brock Street, Kingston, Ontario K7L 5G2 Canada; 3 IC/ES Adjunct Scientist IC/ES Queen’s, Queen’s University, Abramsky Hall, Room 208, 21 Arch Street, Kingston, K7L 3N6 Canada

**Keywords:** Ear, Otology, Undergraduate medical education, Holographic model

## Abstract

**Background:**

Computer-assisted learning has been shown to be an effective means of teaching anatomy, with 3-D visualization technology more successfully improving participants’ factual and spatial knowledge in comparison to traditional methods. To date, however, the effectiveness of teaching ear anatomy using 3-D holographic technology has not been studied. The present study aimed to evaluate the feasibility and effectiveness of learning ear anatomy using a holographic (HG) anatomic model in comparison to didactic lecture (DL) and a computer module (CM).

**Methods:**

A 3-D anatomic model of the middle and inner ear was created and displayed using presentation slides in a lecture, computer module, or via the Microsoft HoloLens. Twenty-nine medical students were randomized to one of the three interventions. All participants underwent assessment of baseline knowledge of ear anatomy. Immediately following each intervention, testing was repeated along with completion of a satisfaction survey.

**Results:**

Baseline test scores did not differ across intervention groups. All groups showed an improvement in anatomic knowledge post-intervention (*p* < 0.001); the improvement was equal across all interventions (*p* = 0.06). Participants rated the interventions equally for delivery of factual content (*p* = 0.96), but rated the HG higher than the DL and CM for overall effectiveness, ability to convey spatial relationships, and for learner engagement and motivation (*p* < 0.001).

**Conclusions:**

These results suggest that 3-D holographic technology is an effective method of teaching ear anatomy as compared to DLs and CMs. Furthermore, it is better at engaging and motivating learners compared to traditional methods, meriting its inclusion as a tool in undergraduate medical education curriculum.

## Introduction

An undergraduate medical curriculum often begins with a foundational course in anatomy, given the importance of this knowledge base for understanding and contextualizing clinical pathophysiology. Didactic lecture and cadaveric dissection have traditionally been the mainstay of anatomical learning, with proponents of its use arguing its importance in a wholistic and multisensory understanding of the human body’s organization, an exposure to anatomic variability, establishing team-based learning, and even fostering a comprehension of patient mortality [10]. However, the challenges to cadaveric dissection include financial costs, time requirements, and limited cadaveric availability. Coupled with the advent of modern computer technologies, this has prompted the exploration of innovative supplemental tools for anatomy education [1, 13, 30].

In addition to simulation and plastinated models, the effectiveness of computer-assisted learning as a means of teaching anatomy has been extensively studied [6, 21]. Computer-based learning is comprised of 2-dimensional and 3-dimensional (3-D) methods. 3-D visualization software efforts have been successful in teaching head and neck [4] and circulation anatomy [32] to undergraduate medical students, in addition to teaching pelvic floor anatomy to more advanced trainees [5]. Comparisons between 2D- and 3D-teaching have been equivocal, with some studies demonstrating no difference between the methods [12, 16], while other studies support the superiority of 3-D technologies [26, 28]. A recent meta-analysis, however, reported that 3-D visualization technologies were overall more effective than 2-D methods in terms of participants’ factual and spatial knowledge acquisition, along with user satisfaction and perception of the learning method [38].

3D-technologies can be further subdivided based on the type of medium employed, namely, tablet displays, virtual reality and augmented reality [23]. 3-D software available for use on smartphones and tablets is becoming increasingly more powerful, such that users can manipulate the view of selected structures and can incrementally add on adjacent structures to suit their own learning pace [20, 31]. Virtual reality technology has expanded with the release of consumer-grade devices such as the Oculus Rift, allowing users to have an immersive educational experience. Examples of such technology include the ability to navigate digital pathology slides [7] and complete a 3-D virtual anatomy puzzle [22]. Augmented reality is another promising technology which involves being able to superimpose digital images and objects on a user’s existing environment. The effectiveness of augmented reality has been demonstrated using handheld mobile devices, resulting in significant improvements in anatomy learning compared to control groups [14]. Interestingly, augmented reality technology has been reported to be less cognitively-demanding for students compared to traditional 2-D methods [17].

The utility of computer-based technology has been studied with regards to teaching ear anatomy. The supplementation of a web-based tutorial with a fully interactive 3-D computer model of the middle and inner ear was found to significantly improve participants’ knowledge compared to the tutorial alone [26, 27]. Active manipulation of a virtual inner ear structure was also reported to promote greater structural recall compared to passive interaction with the model [15]. To date, however, the effectiveness of 3-D holographic models as a means of teaching ear anatomy has not been studied. Furthermore, the middle and inner ear are some of the most spatially complex regions in the body and pose significant challenges to the learner. The present study thus aimed to evaluate the feasibility and effectiveness of learning ear anatomy using holographic (HG) anatomic models in comparison to traditional 2-D learning methods (i.e., didactic lecture [DL] and web-based computer module [CM]).

## Materials and methods

This prospective randomized controlled non-clinical trial was approved by the Queen’s University Health Sciences and Affiliated Teaching Hospitals Research Ethics Board (project #6024569) and the Queen’s University School of Medicine Undergraduate Medical Education Curriculum Committee.

### Participants

Sample size was based on previous work by our research group, [2, 36, 37] which demonstrated adequate power and significant differences using a similar three-intervention design with 8 participants in each group. To account for the possibility of participant withdrawal during the study, we aimed to include 10 participants in each intervention group. All first- and second-year medical students from Queen’s University were invited to participate in the study. Participants were excluded from the analysis if they did not complete the assigned intervention modality along with pre- and post-intervention assessments. After obtaining written consent, participants were randomized to one of the three intervention groups (i.e., DL, CM or HG).

### Didactic lecture (DL)

To standardize the DL intervention, all participants assigned to that arm attended a single lecture taught by the senior author (J.A.B.). The anatomy of the middle and inner ear was explained using various static images obtained from the computer-generated 3-D model used in the HG intervention. Given the short duration of the presentation itself, the lecture was repeated to fill the 30-min allotted time slot.

### Web-based computer module (CM)

Participants randomized to the CM intervention were sequestered for 30 min and allowed to access a time-released module via their personal computers. The module consisted of the same slides used in the presentation to the DL group.

### Holographic model (HG)

**Fig. 1:**
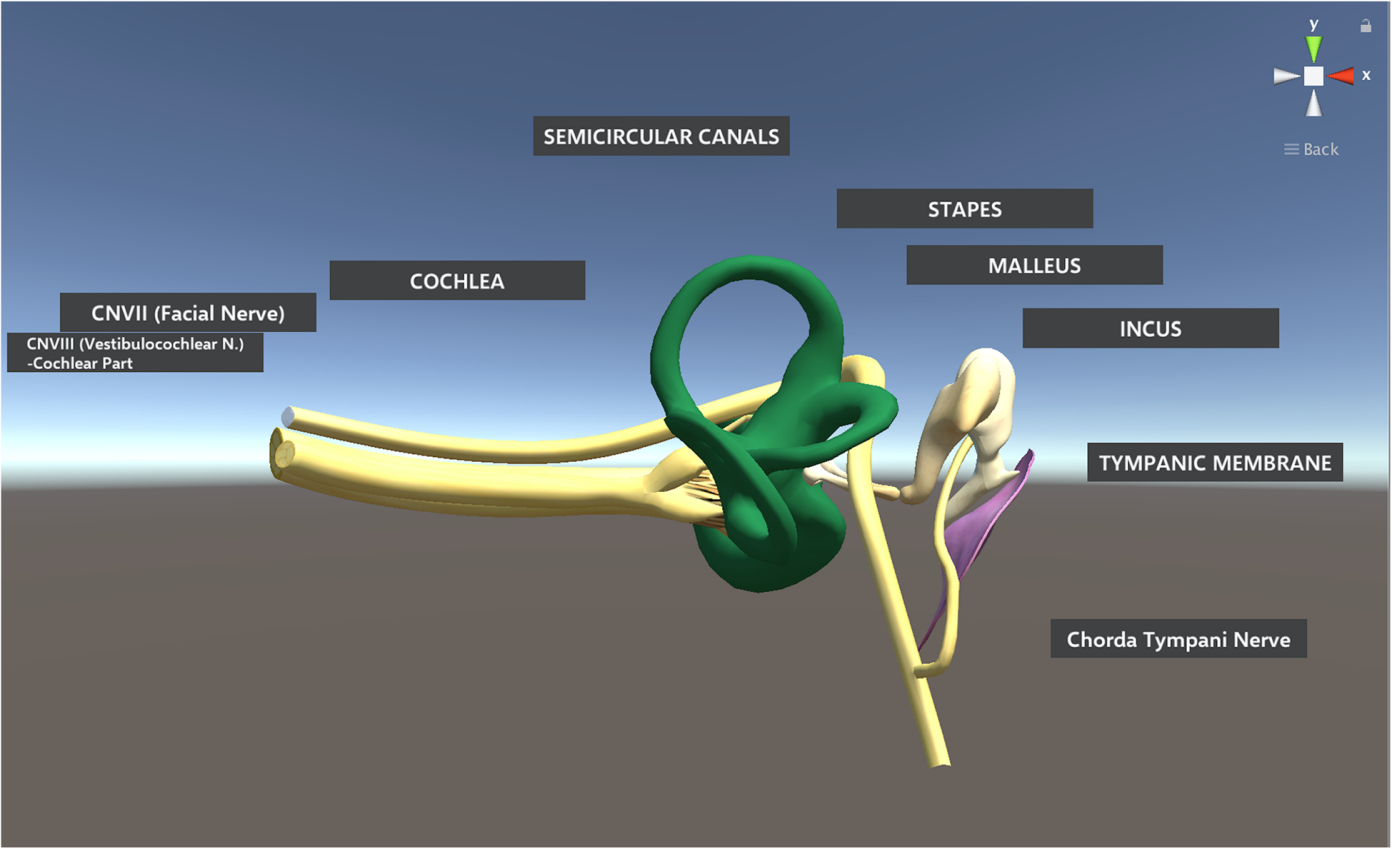
Two-dimensional rendering of the holographic model used to teach anatomy of the middle and inner ear

Object data for the anatomic model used in this study originated from Campbell’s 3-D computer model of the inner ear [3], which in turn, was derived from magnetic resonance images of the human cadaver ear [9, 11]. Both data sets are publicly available and were used under license CC BY-NC-SA 1.0 and CC BY-NC-SA 4.0, respectively. The object data were imported into Unity® software to develop the holographic model used in this study. The model displayed anatomic components of the middle and inner ear and the mastoid structures, namely, the tympanic membrane through the ossicles to the vestibule and cochlea and the associated regional nerves (Fig. 1). All structures were labeled and visible to the user at all times. The model was displayed using the Microsoft HoloLens, which is a head-mounted display unit that uses a pair of transparent combiner lenses to project the images in front of the user. Participants could observe and study the structures at a distance, or choose to walk around the model for spatial exploration as if the model existed in their real physical space. No gesture interactions were implemented for the current model. Prior to beginning this study, this model had been piloted by our research group with another group of 26 undergraduate medical students to ensure the usability of the model.

Since the HG model did not include any text besides the labelled structures, participants in this group were provided with a handout of the text included in the presentation slides of the DL and CM group. Prior to beginning the study, participants in the HG group received an additional 10 min of prior device training to ensure a comfortable fit with the Microsoft HoloLens. The study commenced once participants in this HG arm indicated comfort with seeing and interacting with holographic objects in their space.

### Study design

All participants first completed a baseline assessment test, comprising of 20 multiple-choice questions requiring identification of middle and inner ear anatomical structures. All images used on the test were taken from the 3-D model created for this study. Following the baseline testing, all participants underwent 30 min of training in their corresponding learning intervention arm (DL, CM, or HG, as outlined above). For all three intervention arms, the session instructor (J.J.G.) was immediately available to answer any questions that the participants had.

Upon completion of the intervention, all participants completed the assessment test again. Participants also completed a questionnaire to obtain data on the effectiveness of the learning intervention. The survey comprised of quantitative and qualitative parts, the first of which asked participants to use a 5-point Likert scale (1 = not effective, 5 = very effective) to evaluate their respective learning intervention on the following aspects: (i) overall effectiveness, (ii) ability to teach factual content, (iii) effectiveness of teaching spatial orientation/relationships between anatomic structures, (iv) effectiveness of learner engagement and motivation. In the second part of the survey, participants were asked to name their preferred method of learning (i.e., DL, CM or HG) and explain why they preferred that method. While there was no concerted effort to keep participants blinded as to the other arms of the study, participants were not given details concerning the other teaching modalities until reaching this point in the survey. When answering this question, the other modalities were discussed with participants to help them determine which of the three learning methods they would prefer if all three were available to them. Finally, the survey asked for feedback on how to improve the hologram as a learning tool. Students in the DL and CM groups were not required to answer the last question, however, if they expressed interest in viewing the HG model, they were then able to use the HoloLens device and provide feedback on the model.

### Statistical analysis

SPSS was used for all statistical analyses, with statistical significance set to ∝ = 0.05. Results are reported as mean ± SD. Mixed-design analysis of variance (ANOVA) was used to analyze between-group differences of knowledge acquisition, as measured by assessment test scores, and within-subject differences measured pre- and post-intervention. Bonferroni-corrected *t*-tests were used to compare subgroups post hoc. ANOVAs were used to compare group assessment scores pre- and post-intervention, in addition to participant responses on the satisfaction survey.

## Results

Thirty participants were initially recruited and randomized to an intervention group, with 10 participants each in the DL, CM and HG groups. After randomization, one student in the DL group was unable to attend their intervention session and withdrew. Thus, 29 students ultimately participated in the study, with 10 students each in the CM and HG groups and 9 students participating in the DL group. All students completed the pre- and post-intervention assessments along with the satisfaction survey.

### Knowledge acquisition

**Fig. 2:**
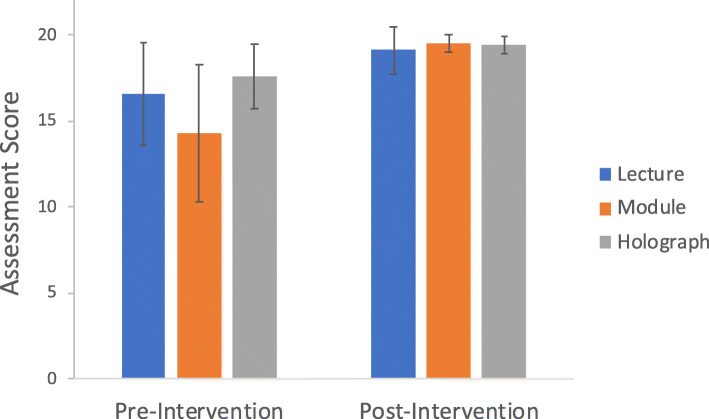
Pre- and post-intervention assessment scores (maximum score of 20) of the Didactic Lecture (DL), web-based Computer Module (CM) and Holographic Model (HG) groups

Average pre- and post-intervention assessment scores (maximum score of 20) are shown in Fig. 2. Pre-intervention assessment scores were 16.6 ± 3.0, 14.3 ± 4.0, and 17.6 ± 1.9 for DL, CM and HG groups, respectively. Post-intervention group scores were 19.1 ± 1.4, 19.5 ± 0.5, 19.4 ± 0.5, respectively. There was no significant difference between the DL, CM and HG groups on the pre-intervention [F_(2,26)_ = 2.99, *p* = 0.07] or the post-intervention [F_(2,26)_ = 0.50, *p* = 0.61] tests. There was a main effect of time (i.e., pre- and post-intervention) on assessment score [F_(1,26)_ = 29.64, *p* < 0.001], given the overall average of the post-intervention test (19.3 ± 0.9) was significantly higher than the pre-intervention test (16.1 ± 3.3; mean difference = 3.2, *p* < 0.001). There was a trending interaction effect between time and the type of intervention (i.e., DL, CM, HG) [F_(2,26)_ = 15.83, *p* = 0.06], given the DL (mean difference = 2.6 ± 3.2, *p* = 0.02) and CM (mean difference = 5.2 ± 3.9, *p* < 0.001) but not the HG (mean difference = 1.8 ± 2.0, *p* = 0.08) groups performed significantly better on the post-intervention assessment compared to the pre-intervention test.

### Satisfaction survey

**Fig. 3:**
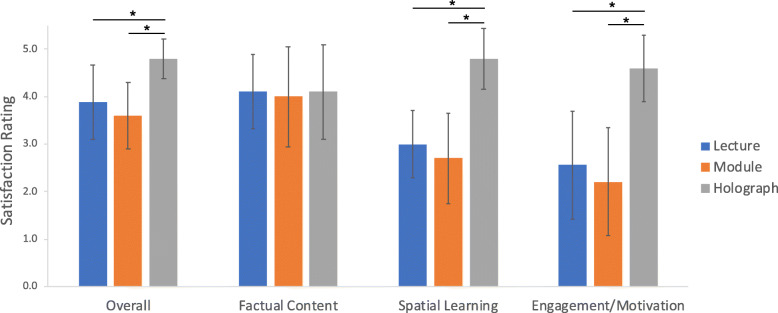
Participants’ ratings of overall effectiveness, factual content delivery, ability to convey spatial relationships between anatomic structures, and ability to keep the learner engaged and motivated

Figure 3 illustrates each participant group’s average rating of only their respective intervention (i.e., DL, CM, HG). There were significant differences in ratings of overall effectiveness [F_(2,28)_ = 9.31, *p* < 0.001], with the HG (4.8 ± 0.4) receiving significantly higher ratings compared to the DL (3.9 ± 0.8, *p* < 0.02) and the CM (3.6 ± 0.7, *p* < 0.001). There was no effect of intervention on ratings of effectiveness at teaching factual content [F_(2,26)_ = 0.04, *p* = 0.96]. Participants’ ratings of the teaching modalities significantly differed in terms of effectiveness of conveying spatial relationships between anatomical structures [F_(2,26)_ = 21.15, *p* < 0.001], with the HG (4.8 ± 0.6) being rated significantly higher than the DL (3.0 ± 0.7, *p* < 0.001) and the CM (2.7 ± 0.9, *p* < 0.001). There were significant differences between participants’ ratings of the teaching modality’s learner engagement and motivation [F_(2,26)_ = 16.47, *p* < 0.001], such that the HG (4.6 ± 0.7) was rated higher than the DL (2.6 ± 1.1, *p* < 0.001) and the CM (2.2 ± 1.1, *p* < 0.001).

**Fig. 4:**
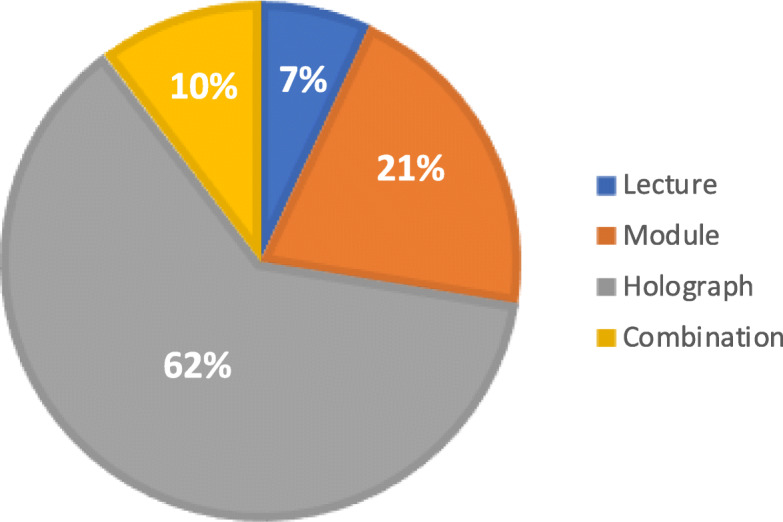
Students’ preference of teaching modality

As Fig. 4 illustrates, when asked to indicate a preferred modality of learning anatomy, 2 (7%) participants indicated DL, 6 (21%) chose CM, 18 (62%) selected HG, and 3 (10%) preferred a combination of CM and HG [χ^2^_(3, *N* = 29)_ = 22.45, *p* < 0.001].

## Discussion

This prospective randomized controlled non-clinical trial was a proof-of-concept study aimed at evaluating the feasibility and effectiveness of learning ear anatomy using holographic anatomical models in comparison to traditional 2-D learning methods (i.e., didactic learning and web-based learning module). The primary outcome was factual and spatial knowledge acquisition of anatomic structures of the middle and inner ear, as demonstrated by quantitative tests administered at baseline and post-intervention. Secondary outcomes included learner interest and overall perception of the learning method, as indicated by qualitative questionnaires completed by all participants.

There was no significant difference between baseline knowledge of ear anatomy between the DL, CM and HG groups, confirming adequate randomization of participants prior to commencing the study. The current pre-clerkship curriculum at Queen’s University utilizes didactic lectures, small group learning and cadaveric specimens to teach medical students the anatomy (macroscopic and histologic) and physiology of the outer, middle and inner ear. Furthermore, students are taught the various clinical presentations that are consequences of pathophysiological processes negatively impacting these otologic structures. The extensive curricular time spent on otologic anatomy and pathophysiology may perhaps help explain why participants performed well, on average, on the pre-intervention assessment.

All three groups demonstrated a consistent improvement in factual and spatial knowledge after completing their respective learning interventions. This improvement in factual knowledge is congruent with numerous studies that have successfully used computer-based technologies to teach anatomy [5, 18, 33, 34]. While augmented reality technology has been used to teach various components including skull anatomy [23], neuroanatomy [17], and skeletal structure [14], this is the first study to date to apply 3-D holographic technology towards middle and inner ear anatomy, demonstrating its feasibility and effectiveness in this domain as well.

There were no significant differences noted among the three groups on post-intervention assessment scores, indicating that all methods of teaching ear anatomy were similarly effective. The finding of equal effectiveness among the three teaching modalities (DL, CM and HG) was surprising, given that a meta-analysis of 28 studies found that in comparison to other 2-D of teaching, 3-D visualization technologies resulted in higher factual knowledge and spatial knowledge acquisition [38]. Previous work by our group has investigated the effect of teaching modality on students’ ability to correctly diagnose middle and external ear pathology and learn otoscopy skills. When pre- and post-intervention assessments were conducted on an otoscopy simulator, post-intervention diagnostic accuracy was higher in the groups taught with a simulator and CM compared to a DL [36]. When pathology assessments were conducted using real patients, however, there were no significant differences on post-intervention diagnostic accuracy between the simulator-, CM- and DL-taught groups [37]. One possible explanation, then, is that while 3-D technologies are overall more effective at teaching anatomy compared to other methods [38], the particular method of assessment used in a study may determine the relative effectiveness of one teaching modality compared to the others. In comparison to the traditional 2-D assessment, a 3-D-based assessment (e.g., anatomy “bell-ringer”) might be better suited to capture knowledge differences between the HG and DL/CM interventions, the former of which presumably better teach anatomical spatial relationships due to the nature of the respective intervention compared to the 2-D DL and CM methods. Alternatively, the assessment questions may not have been sufficiently difficult, thus ceiling effects may have been reached with regards to improvements on the tests. Including more advanced content on the model and providing a more rigorous assessment of factual and spatial knowledge would perhaps elucidate clear score differences between the groups.

Significant differences emerged when participants were asked to provide an overall rating of their respective teaching modality, with students in the HG group rating their intervention significantly higher than the DL and CM groups. While the groups indicated there was no difference in the effectiveness at which each modality presented the factual content, participants in the HG group were more satisfied with how spatial relationships between anatomical structures were shown as compared to participants in either the DL or CM groups. Furthermore, the HG was rated higher by participants on stimulating learner engagement and motivation compared to the other two teaching modalities, such that when finally asked which method they would have preferred, 62% of participants chose the HG, regardless of intervention arm they had been placed in. The lack of a unanimous preference for the HG suggests that students were not merely opting for a novel technology, but instead, thoughtfully selecting the device for the perceived improved learning experience. This student preference for 3-D learning is a recurrent theme reported in anatomy pedagogical literature [12, 35, 38]. Given the motivational incentive that it provides to learners, holographic and augmented reality technology can be a powerful tool when used as an adjunct to DL or CM [24, 25, 27], particularly because 3-D technologies have been shown to equip trainees with a better understanding of anatomic spatial relationships compared to 2-D methods [29].

Given the proof-of-concept nature of this study, the holographic model used has room for further improvement. In fact when asked for written feedback about the model, participants indicated they would like to see more complex anatomy in the future, including the option to layer adjacent anatomical structures in a step-wise function, so as to be able to contextualize the illustrated components. Participants additionally suggested designing the model so that it was not fixed in one place but could instead be manipulated by the user. This added feature may enhance factual and spatial knowledge retention, as the direct manipulation of virtual 3-D structures has been shown to improve the fidelity of users’ internal representation of anatomical structures with regards to shape, location and orientation [15, 19]. In addition, participants requested the integration of text into the model itself, such that the user could select an anatomical structure, and a pop-up text block would appear that described the selected portion and outlined its functional role. Another suggestion was to program animations into the model (e.g., the conductance of sound waves from the tympanic membrane and ossicles into the cochlea), allowing students to overlay relevant physiology on the displayed structures. Our group will pursue these suggestions going forward.

In addition to the aforementioned simplicity of model design, a limitation of the study includes a lack of long-term participant follow-up to ascertain long-term knowledge retention. When the long-term (6-month) retention of laryngeal anatomy was assessed in students who had learned either from standard written instruction or a 3-D computer model, no significant difference in scores was noted [8]. In contrast, the integration of varying learning methods appears to promote long-term retention of neuroanatomy [25]. The present study included second-year medical students as participants, who would be in clerkship at the typical 6-month and 1-year follow-up time points. As such, a significant loss to follow-up was predicted for this cohort. Future studies by our group could include a longitudinal component by focusing on first year medical learners, who could then be followed more closely during their pre-clerkship curriculum, or by switching to online assessment methods to facilitate participants’ remote completion of the tests. As a number of participants indicated a preference for combined CM- and HG-learning, future studies should also be directed at examining the effect of multi-modal teaching on immediate factual and spatial acquisition and retention.

## Conclusions

The results of this study demonstrate that 3-D holographic technology is an effective method of teaching ear anatomy as compared to didactic lectures and computer-based modules. Furthermore, 3-D holographic technology is well-received by students, more effectively conveys complex spatial relationships between anatomic structures, and is better at engaging and motivating learners as compared to traditional 2-D methods of teaching. The authors believe that 3-D holographic technologies are useful tools that merit inclusion in Otolaryngology – Head and Neck Surgery undergraduate medical education curriculum. Given the financial yearly cost and limited availability of cadaveric dissection, it is plausible that fully-developed augmented reality models could, in the future, serve as a more economically sustainable alternative or supplement to this traditional method of teaching anatomy.

## Supplementary information

**Additional file 1.**

## Data Availability

Object data for the anatomic model used in this study originated from Campbell’s 3-D computer model of the inner ear [3], which in turn, was derived from magnetic resonance images of the human cadaver ear [9, 11]. Both data sets are publicly available and were used under license CC BY-NC-SA 1.0 and CC BY-NC-SA 4.0, respectively. The datasets generated during and/or analysed during the current study are available from the corresponding author on reasonable request.

## Abbreviations

3-D: Three dimensional
ANOVA: Analysis of variance
CM: Computer Module
DL: Didactic lecture
HG: Holographic

## References

[1] Bergman EM (2015). Discussing dissection in anatomy education. Perspect Med Educ.

[2] Beyea JA, Wong E, Bromwich M, Weston WW, Fung K (2008). Evaluation of a particle repositioning maneuver web-based teaching module. Laryngoscope..

[3] Campbell A (2016). Anatomy of the Inner Ear.

[4] Cui D, Wilson TD, Rockhold RW, Lehman MN, Lynch JC (2017). Evaluation of the effectiveness of 3D vascular stereoscopic models in anatomy instruction for first year medical students. Anat Sci Educ.

[5] Dobson HD, Pearl RK, Orsay CP, Rasmussen M, Evenhouse R, Ai Z, Blew G, Dech F, Edison MI, Silverstein JC, Abcarian H (2003). Virtual reality: new method of teaching anorectal and pelvic floor anatomy. Dis Colon Rectum.

[6] Estai M, Bunt S (2016). Best teaching practices in anatomy education. Ann Anat.

[7] Farahani N, Post R, Duboy J, Ahmed I, Kolowitz BJ (2016). Exploring virtual reality technology and the oculus rift for the examination of digital pathology slides. J Pathol Inform.

[8] Fritz D, Hu A, Wilson T, Ladak H, Haase P, Fung K (2011). Long-term retention of a 3-dimensional educational computer model of the larynx: a follow-up study. Arch Otolaryngol Head Neck Surg.

[9] Funnell RJ, Daniel S, Nicholson D (2006). 3D ear.

[10] Granger NA (2004). Dissection laboratory is vital to medical gross anatomy education. Anat Rec B New Anat.

[11] Henson OW Jr, and Henson M. The Vertebrate Ear and Temporal Bone.Retrieved from http://cbaweb2.med.unc.edu/henson_mrm/.

[12] Hu A, Wilson T, Ladak H, Haase P, Doyle P, Fung K (2010). Evaluation of a three-dimensional educational computer model of the larynx: voicing a new direction. J Otolaryngol Head Neck Surg.

[13] Hu M, Wattchow D, de Fontgalland D (2018). From ancient to avant-Garde: a review of traditional and modern multimodal approaches to surgical anatomy education. ANZ J Surg.

[14] Jamali SS, Shiratuddin MF, Wong KW, Oskam CL (2015). Utilising mobile-augmented reality for learning human anatomy. Procedia..

[15] Jang S, Vitale JM, Jyung RW, Black JB (2017). Direct manipulation is better than passive viewing for learning anatomy in a three-dimensional virtual reality environment. Comput Educ.

[16] Keedy AW, Durack JC, Sandhu P, Chen EM, O’Sullivan PS, Breiman RS (2011). Comparison of traditional methods with 3D computer models in the instruction of hepatobiliary anatomy. Anat Sci Educ.

[17] Kucuk S, Kapakin S, Goktas Y (2016). Learning anatomy via mobile augmented reality: effects on achievement and cognitive load. Anat Sci Educ.

[18] Kuszyk BS, Calhoun PS, Soyer PA, Fishman EK (1997). An interactive computer-based tool for teaching the segmental anatomy of the liver: usefulness in the education of residents and fellows. AJR Am J Roentgenol.

[19] Lages W, Bowman D (2018). Move the object or move myself? Walking vs. manipulation for the examination of 3D scientific data. Front ICT.

[20] Lewis TL, Burnett B, Rg T, Abrahams PH (2014). Complementing anatomy education using three-dimensional anatomy mobile software applications on tablet computers. Clin Anat.

[21] Losco CD, Grant WD, Armson A, Meyer AJ, Walker BF (2017). Effective methods of teaching and learning in anatomy as a basic science: a BEME systematic review: BEME guide no. 44. Med Teach..

[22] Messier E, Wilcox J, Dawson-Elli A, Diaz G, Linte CA (2016). An interactive 3D virtual anatomy puzzle for learning and simulation – initial demonstration and evaluation. Stud Health Technol Inform.

[23] Moro C, Stromberga Z, Raikos A, Stirling A (2017). The effectiveness of virtual and augmented reality in health sciences and medical anatomy. Anat Sci Educ.

[24] Murgitroyd E, Madurska M, Gonzalez J, Watson A (2015). 3D digital anatomy modelling – practical or pretty?. Surgeon..

[25] Naaz F, Chariker JH, Pani JR (2014). Computer-based learning: graphical integration of whole and sectional neuroanatomy improves long-term retention. Cogn Instr.

[26] Ng CL, Liu X, Chee SC, Ngo RY (2015). An innovative 3-dimensional model of the epitympanum for teaching of middle ear anatomy. Otolaryngol Head Neck Surg..

[27] Nicholson DT, Chalk C, Funnell WR, Daniel SJ (2006). Can virtual reality improve anatomy education? A randomised controlled study of a computer generated three-dimensional anatomical ear model. Med Educ.

[28] Nickel F, Hendrie JD, Bruckner T, Kowalewski KF, Kenngott HG, Muller-Stich BP, Fischer L (2016). Successful learning of surgical liver anatomy in a computer-based teaching module. Int J Comput Assist Radiol Surg.

[29] Pahuta MA, Schemitsch EH, Backstein D, Papp S, Gofton W (2012). Virtual fracture carving improves understanding of a complex fracture: a randomized controlled study. J Bone Joint Surg Am.

[30] Papa V, Vaccarezza M (2013). Teaching anatomy in the XXI century: new aspects and pitfalls.

[31] Park S, Kim Y, Park S, Shin JA (2019). The impacts of three-dimensional anatomical atlas on learning anatomy. Anat Cell Bio.

[32] Petersson H, Sinkvist D, Wang C, Smedby O (2009). Web-based interactive 3D visualization as a tool for improved anatomy learning. Anat Sci Educ.

[33] Silverstein JC, Dech F, Edison M, Jurek P, Helton WS, Espat NJ (2002). Virtual reality: immersive hepatic surgery educational environment. Surgery..

[34] Venail D, Deveze A, Lallemant B, Guevara N, Mondain M (2010). Enhancement of temporal bone anatomy learning with computer 3D rendered imaging software. Med Teach.

[35] Wright SJ (2012). Student perceptions of an upper-level, undergraduate human anatomy laboratory course without cadavers. Anat Sci Educ.

[36] Wu V, Beyea JA (2017). Evaluation of a web-based module and an Otoscopy simulator in teaching ear disease. Otolaryngol Head Neck Surg.

[37] Wu V, Sattar J, Cheon S, Beyea JA (2018). Ear disease knowledge and Otoscopy skills transfer to real patients: a randomized controlled trial. J Surg Educ.

[38] Yammine K, Violato C (2015). A meta-analysis of the educational effectiveness of three-dimensional visualization technologies in teaching anatomy. Anat Sci Educ.

