# Supplementary material for: Evaluating the effectiveness of learning ear anatomy using holographic models
Source: J Otolaryngol Head Neck Surg. 2020 Aug 19;49:63. doi: 10.1186/s40463-020-00458-x (PMC7439621; doi:10.1186/s40463-020-00458-x)
Supplement: Supplementary file 1 — Additional file 1. [file 40463_2020_458_MOESM1_ESM.pdf]

Evaluating the Effectiveness of Learning Ear Anatomy Using Holographic Models

Investigators: Joshua Gnanasegaram, Regina Leung
Principal Investigator: Jason Beyea, MD PhD FRCSC

Participant ID:
Date:

1. The order of ossicles, from lateral to medial is:
    a. Incus, malleus, stapes
    b. Stapes, incus, malleus
    c. Malleus, incus, stapes
    d. Malleus, stapes, incus

2. The chorda tympani is a branch of which cranial nerve?
    a. CN VI
    b. CN VII
    c. CN VIII
    d. CN IX

3. After entering the tympanic cavity, the chorda tympani passes between which two structures?
    a. Malleus and incus
    b. Stapes and cochlea
    c. Facial and vestibulocochlear nerves
    d. Incus and stapes

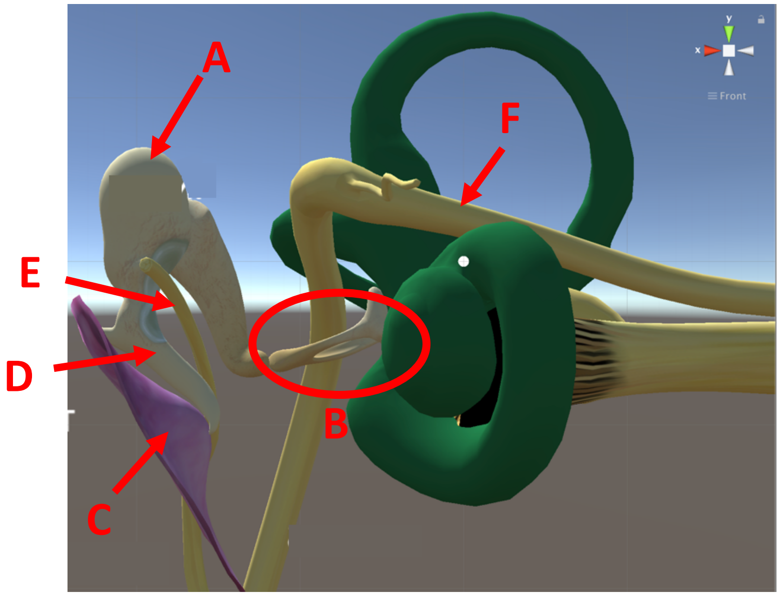

Queen's University

4. The structure marked “A” in the picture above is called the…
    a. Stapes
    b. Malleus
    c. Incus
    d. Tympanic membrane

5. The circled structure marked “B” in the picture above is called the…
    a. Stapes
    b. Malleus
    c. Incus
    d. Tympanic membrane

6. The structure marked “C” in the picture above is called the…
    a. Stapes
    b. Malleus
    c. Incus
    d. Tympanic membrane

7. The structure marked “D” in the picture above is called the…
    a. Stapes
    b. Malleus
    c. Incus
    d. Tympanic membrane

8. The structure marked “E” in the picture above is called the…
    a. Facial nerve
    b. Cochlear nerve
    c. Chorda Tympani
    d. Vestibular nerve

9. The structure marked “F” in the picture above is called the…
    a. Facial nerve
    b. Cochlear nerve
    c. Chorda Tympani
    d. Vestibular nerve

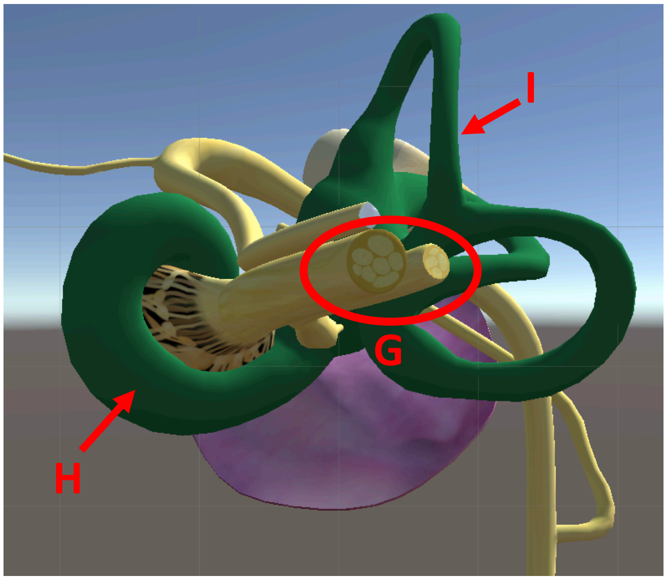

10. The structures marked “G” in the picture above make up the…
    a. Facial nerve
    b. Chorda Tympani
    c. Cochleovestibular nerve
    d. Trigeminal nerve

11. The structure marked “H” in the picture above is called the…
    a. Tympanic membrane
    b. Otoliths
    c. Cochlea
    d. Semicircular canals

12. The structure marked “I” in the picture above is called the…
    a. Tympanic membrane
    b. Otoliths
    c. Cochlea
    d. Semicircular canals

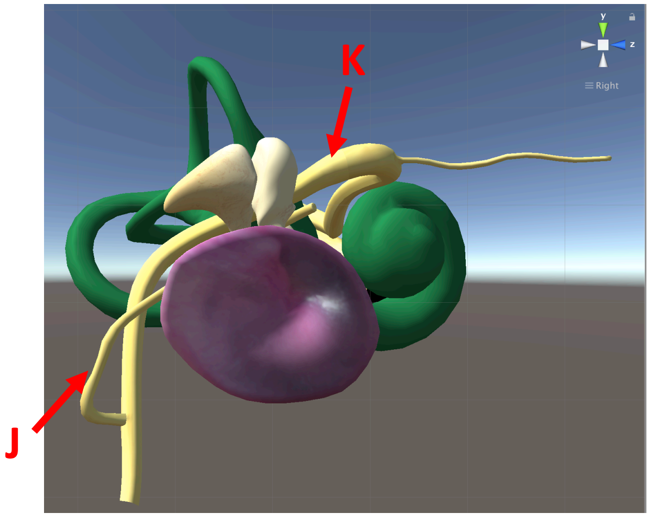

13. The structure marked “J” in the picture above is called the…
    a. Facial nerve
    b. Chorda Tympani
    c. Cochleovestibular nerve
    d. Trigeminal nerve
14. The structure marked “K” in the picture above is called the…
    a. Facial nerve
    b. Chorda Tympani
    c. Cochleovestibular nerve
    d. Trigeminal nerve

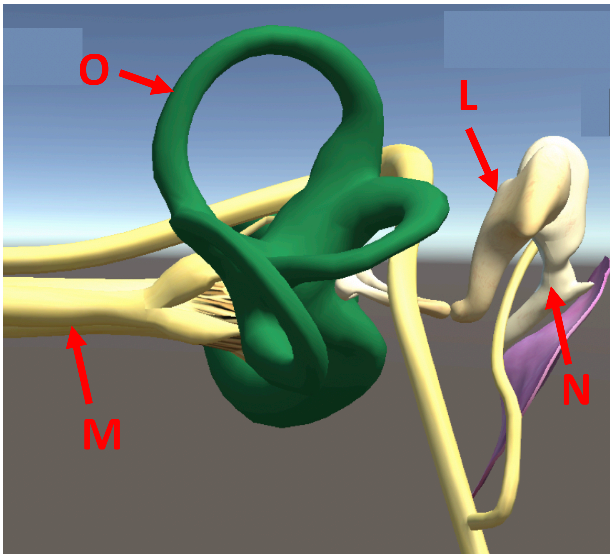

15. The structure marked “L” in the picture above is called the…
    a. Stapes
    b. Malleus
    c. Incus
    d. Tympanic membrane

16. The structure marked “M” in the picture above is called the…
    a. Facial nerve
    b. Chorda Tympani
    c. Cochleovestibular nerve
    d. Trigeminal nerve

17. The structure marked “N” in the picture above is called the…
    a. Stapes
    b. Malleus
    c. Incus
    d. Tympanic membrane

18. The structure marked “O” in the picture above is called the…
    a. Tympanic membrane
    b. Otoliths
    c. Cochlea
    d. Semicircular canals

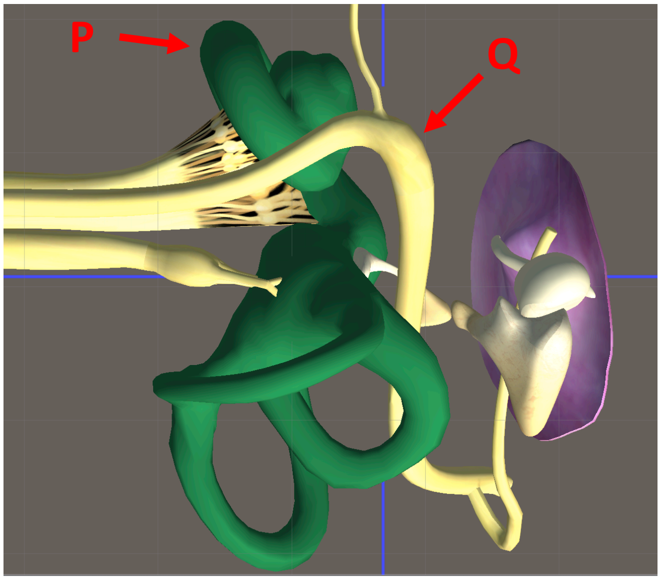

19. The structure marked “P” in the picture above is called the…
    a. Tympanic membrane
    b. Otoliths
    c. Cochlea
    d. Semicircular canals

20. The structure marked “Q” in the picture above is called the…
    a. Facial nerve
    b. Chorda Tympani
    c. Cochleovestibular nerve
    d. Trigeminal nerve
